# Full-Color See-Through Three-Dimensional Display Method Based on Volume Holography

**DOI:** 10.3390/s21082698

**Published:** 2021-04-11

**Authors:** Taihui Wu, Jianshe Ma, Chengchen Wang, Haibei Wang, Ping Su

**Affiliations:** 1 Department of Precision Instrument, Tsinghua University, Beijing 100084, China; wuth18@mails.tsinghua.edu.cn (T.W.); chengche19@mails.tsinghua.edu.cn (C.W.); 2 Tsinghua Shenzhen International Graduate School, Tsinghua University, Shenzhen 518055, China; ma.jianshe@sz.tsinghua.edu.cn (J.M.); whb20@mails.tsinghua.edu.cn (H.W.)

**Keywords:** holographic display, volume holography, photopolymer, augmented reality

## Abstract

We propose a full-color see-through three-dimensional (3D) display method based on volume holography. This method is based on real object interference, avoiding the device limitation of spatial light modulator (SLM). The volume holography has a slim and compact structure, which realizes 3D display through one single layer of photopolymer. We analyzed the recording mechanism of volume holographic gratings, diffraction characteristics, and influencing factors of refractive index modulation through Kogelnik’s coupled-wave theory and the monomer diffusion model of photopolymer. We built a multiplexing full-color reflective volume holographic recording optical system and conducted simultaneous exposure experiment. Under the illumination of white light, full-color 3D image can be reconstructed. Experimental results show that the average diffraction efficiency is about 53%, and the grating fringe pitch is less than 0.3 μm. The reconstructed image of volume holography has high diffraction efficiency, high resolution, strong stereo perception, and large observing angle, which provides a technical reference for augmented reality.

## 1. Introduction

When the movie Avatar was released in 2009, people were deeply impressed by the 3D display. With the vigorous development of augmented reality (AR), new application scenarios have been brought to 3D display. Augmented reality has been widely used in medical, education, industry, entertainment, and construction fields [1,2]. Head-mounted displays (HMDs) are common AR devices. However, many HMDs are based on the stereoscopic display technology of binocular parallax, which belongs to pseudo 3D display and cannot completely reproduce the continuous wavefront information of the object. In addition, binocular parallax causes the problem of convergence-accommodation conflicts, which can easily cause visual fatigue [3,4]. Among various 3D display technologies, holography can reconstruct the amplitude and phase information required by the human eye with high quality, which has become an important research hotspot in AR display field. The holographic 3D display records the wavefront information of the object through the interference, and realizes the reproduction of the object by illuminating the hologram. This technology can retain the depth information of the object and is considered to be one of the most promising 3D display technologies.

Currently, holographic 3D displays have some typical problems, such as speckle noise, resolution and spatial bandwidth product limitations, and so on. In addition, due to the small diffraction angle and the number of pixels of the spatial light modulator (SLM), the observing angle and image size of the holographic 3D display are limited. Large perspective and full-color display are important technical difficulties for current holographic display. In response to these problems, many researchers have proposed different 3D holographic display solutions. Juan Liu et al. [5] proposed a full-color transparent near-eye holographic display, which has an 80° field of view and an extended eye box. The system has a large field of view and eliminates crosstalk. Hongyue Gao et al. [6] proposed a holographic 3D virtual reality and augmented reality display method based on a 4K SLM. The viewing angle of the display was expanded from 3.8° to 16.4° through SLM splicing. Gang Li et al. [7] used holographic optical elements (HOE) to realize a see-through augmented reality holographic display, which has the functions of a mirror and a lens at the same time. Koki Wakunami et al. [8] proposed a projection-type see-through holographic 3D display, HOE produced by this method has a wide range of application potential in smart glasses, HMD, and vehicle displays. Seung-Ho Shin et al. [9] proposed a coherent 3D display method through optical refraction volume holographic storage and integral imaging, which effectively reduces speckle noise. Takeshi Yamaguchi et al. [10] designed a volume holographic printer to record 3D object images, segment 3D objects through multiple computer-generated holograms, and use 4f optical system to improve image reconstruction quality. Keehoon Hong et al. [11] proposed a hogel overlay method to enhance the lateral resolution of holographic stereograms. Jinyoung Roh et al. [12] designed a full-color holographic projection display system using an achromatic Fourier filter. Zhenxiang Zeng et al. [13] designed a full-color holographic display that uses a layered Fresnel diffraction algorithm to generate RGB (Red, Green, Blue) phase-only holograms. Mei-lan Piao et al. [14] proposed a full-color holographic diffuser with time-sharing and iterative exposure. This method is based on the wavelength multiplexing characteristics of volume holographic gratings. The three-wavelength hologram is recorded on a holographic dry plate to achieve a specific color balance of the multicolor holographic diffuser.

In this research, we propose a full-color see-through 3D display method based on volume holography. Since the diffraction angle and the number of pixels of the SLM are small, we use real object for interference recording. This approach avoids the device limitations caused by SLM, such as the small diffraction angle and the number of pixels. We use the simplified holographic grating diffusion model to analyze the influencing factors of refractive index modulation, including exposure intensity, exposure energy and diffusion time constant. Subsequently, the transmission spectrum and diffraction efficiency of the hologram were analyzed through experiments. Finally, the RGB multiplexing reflective volume holography recording optical system was built. Experimental results show that the RGB multiplexing optical setup can record full-color 3D image by only one single layer of photopolymer. The thickness of the photopolymer is about 16 μm, which can be applied to the next generation of integrated displays. The reconstructed image of volume holography has high resolution, strong stereoscopic effect and large observing angle, and realizes full-color 3D display.

The paper is arranged as follows: In Section 2, related theories are introduced, including Bragg’s law, Kogelnik’s coupled wave theory and the monomer diffusion model. In Section 3, the transmission spectrum and diffraction efficiency of the photopolymer were tested through experiments. In the Section 4, the RGB multiplexing full-color reflective volume holographic recording optical setup is built, and the simultaneous exposure experiment were carried out. Section 5 is a summary of the full text.

## 2. Design Theory and Method

### 2.1. Coupled Wave Theory

Volume holographic gratings can reproduce the wavefront information of real 3D object. The principle of volume holographic 3D display is based on the interference, and the diffraction phenomenon of volume holography occurs under the irradiation of reproduced light. When the grating constant, the Bragg angle and the wavelength of the reproduced light satisfy Bragg’s law, the volume holographic grating exhibits the strongest diffraction efficiency. The equation of Bragg’s law is as follows [15]:

(1) $$2n\Lambda \sin\theta_{0}=\lambda ,$$

(2) $$SF=\frac{1}{\Lambda },$$

where Λ represents the grating constant, and $\theta_{0}$ represents the Bragg angle. λ is the wavelength of reproduced beam in vacuum. *n* represents the refractive index of the medium. *SF* represents the grating spatial frequency. It can be known from Bragg’s law that when the Bragg angle is 90°, the grating spatial frequency is the largest. In this case, the angle between the reference light and the object light is 180°, and the plane of the recording medium bisects the reference light and the object light vertically.

Kogelnik’s coupled wave theory reveals the influence of factors on the diffraction efficiency in volume holography. The diffraction efficiency of non-absorbent reflective volume holographic gratings can be calculated according to the coupled wave theory [16]:

(3) $$\eta_{R}=\frac{{\mathrm{sh}}^{2}\sqrt{\nu^{2}-\xi^{2}}}{{\mathrm{sh}}^{2}\sqrt{\nu^{2}-\xi^{2}}+(1-\frac{\xi^{2}}{\nu^{2}})},$$

(4) $$\nu =\frac{\pi d\Delta n}{\lambda \sqrt{\cos\theta_{r}\cos\theta_{s}}},$$

(5) $$\xi =kd\Delta \theta \sin\left(\phi -\theta_{0}\right)/2\cos\theta_{s}-k^{2}d\Delta \lambda /8\pi n\cos\theta_{s},$$

where $\eta_{R}$ represents the diffraction efficiency of the non-absorption reflective volume holographic grating. n is the refractive index of the medium. λ is the wavelength of incident light. v represents the coupling strength of the volume holographic grating. ξ represents the Bragg mismatch. d is the thickness of the recording medium. Δn represents the refractive index modulation. $\theta_{r}$ is the angle between the reproduced light wave and the *Z* axis in the dielectric material. $\theta_{s}$ is the angle between the diffracted light wave and the *Z* axis in the dielectric material. Δθ is the angular offset. Δλ is the wavelength shift. k=2π/Λ , which represents the size of the grating vector. ϕ is the tilt angle of the grating vector. Sorting out the above equations, the refractive index modulation of the volume holographic grating when the Bragg condition is satisfied can be calculated by the diffraction efficiency:

(6) $$\Delta n=\frac{\lambda \mathrm{arctanh}(\sqrt{\eta_{R}})\sqrt{\cos\theta_{r}\cos\theta_{s}}}{\pi d},$$

**Figure 1:**
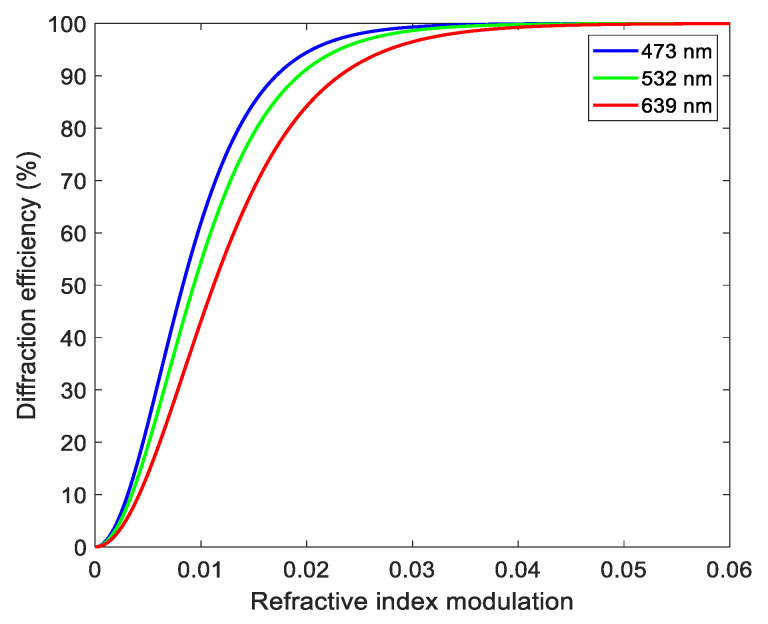
Reflective volume holographic grating: the relationship between refractive index modulation and diffraction efficiency.

Setting the angle between the reference light and the object light is 180° and the thickness of the recording medium is 16 μm, we simulated the relationship between the refractive index modulation and the diffraction efficiency in the reflector holographic grating. As shown in Figure 1, the diffraction efficiency increases with the increase in refractive index modulation, and the growth rate gradually tends to be flat. When the refractive index modulation is greater than 0.01, the diffraction efficiency of each wavelength exceeds 40%. When the refractive index modulation is greater than 0.04, the diffraction efficiency of each wavelength is close to 100%. In order to improve the diffraction efficiency of volume holographic gratings, it is necessary to increase the refractive index modulation as much as possible. Therefore, it is necessary to analyze the influencing factors of the refractive index modulation of the volume holographic grating. Next, we will analyze the refractive index modulation through the monomer diffusion model of the holographic grating.

### 2.2. Monomer Diffusion Model

Photopolymer does not require any chemical or heat treatment [17], and can perform RGB full-color and depth recording, with low detuning, small shrinkage, high resolution, and high diffraction efficiency. These excellent properties have made photopolymers receive more and more attentions. In this study, photopolymer was used as the recording medium of volume holographic grating. The photopolymer is a type of free radical polymerization, and during interference exposure, a photochemical reaction occurs inside it. Holographic exposure results in the formation of light and dark interference fringes in the recording area. The monomer molecules are polymerized to form polymer chains, which reduces the monomer concentration in the recording area. Since the monomer polymerization speed in the bright stripe area is faster, the monomer concentration in the bright stripe area is lower than that in the dark stripe area. A concentration gradient is formed between the dark area and the bright area. Under the effect of the distribution gradient, the monomer molecules continuously diffuse from the dark stripe area to the light stripe area. The above process leads to changes in the refractive index of different regions in the photopolymer, resulting in refractive index modulation, and finally forming a volume holographic diffraction phenomenon. In order to describe the above process, in 1994, Zhao proposed a classic photopolymer grating diffusion model [18]:

(7) $$\frac{\partial u(x,t)}{\partial t}=\frac{\partial }{\partial x}\left[D(x,t)\frac{\partial u(x,t)}{\partial x}\right]-F(x,t)u(x,t),$$

wherein u(x,t) represents the monomer concentration, F(x,t) represents the polymerization rate, and D(x,t) represents the diffusion coefficient. Under the illumination of interference of the object and reference waves in holographic exposure, the monomer and polymer present a periodic distribution in the material. Therefore, the monomer concentration can be expressed by Fourier series. The change of the diffusion coefficient is determined by the distribution of the polymer in the medium, so the diffusion coefficient can also be expressed by a Fourier series. The expressions of diffusion coefficient and monomer concentration by Fourier series are

(8) $$D(x,t)=\sum_{i=0}^{\infty }D_{i}(t)\cos(iKx),$$

(9) $$u(x,t)=\sum_{i=0}^{\infty }u_{i}(t)\cos(iKx),$$

According to Equations (7)–(9), a series of differential equations can be obtained by separating the variables, and Zhao conducted numerical simulations based on this. The simulation results show that during the formation of volume holographic gratings, the diffusion coefficient is mainly determined by the zero-order term in the Fourier series expansion, and the monomer concentration is mainly determined by the first two terms of the Fourier series expansion. Therefore, the diffusion coefficient can be approximately expressed as a constant.

However, Zhao finally did not give an analytical solution to the monomer diffusion model. Subsequently, Pengfei Liu et al. [19] established a simplified diffusion model of the formation process of holographic gratings based on the Zhao diffusion model. This model comprehensively considers the influence of monomer polymerization and diffusion on the refractive index of the dielectric material, and finally obtains the refractive index modulation of the volume holographic grating [19]:

(10) $$\Delta n(t)=mC_{n}U\left\{-\delta \exp\left(-I_{0}^{\delta }\kappa t\right)+\delta \frac{1+\kappa I_{0}^{\delta }\tau \exp\left[-\frac{\left(\kappa I_{0}^{\delta }\tau +1\right)t}{\tau }\right]}{\left(1+\kappa I_{0}^{\delta }\tau \right)}\right\},$$

wherein $C_{n}$ is the proportional coefficient. m is the degree of modulation of interference fringes. U is the initial monomer concentration. δ is the order of magnitude of light response. $I_{0}$ is the exposure intensity. $\tau =1/Dk^{2}$ represents the diffusion time constant. *D* is the diffusion coefficient and κ is the polymerization coefficient, which are constants related to the material. When t≫τ , that is, when the recording time is long enough, the above equation is simplified, and the saturation refractive index modulation of the material can be obtained:

(11) $$\Delta n_{SAT}=\frac{mC_{n}U\delta }{\left(\kappa I_{0}^{\delta }\tau +1\right)}.$$

**Figure 2:**
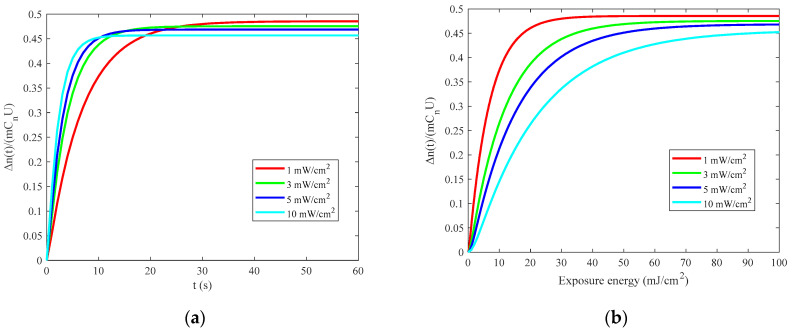
Analysis of factors affecting the refractive index modulation: (**a**) exposure time; (**b**) exposure energy.

According to the theory of radical polymerization [20,21], we set δ as 1/2. It can be seen from this equation that the main factors affecting the saturation refractive index modulation are exposure light intensity and diffusion time constant. Through the numerical simulation of the equations, the qualitative influence of the two parameters on the refractive index modulation is analyzed. Since $mC_{n}U$ is a constant in the equation, $\Delta n\left(t\right)/\left(C_{n}mU\right)$ is selected as the ordinate, and the exposure energy is the abscissa. In the numerical simulation, we set the polymerization coefficient $\kappa =0.15\;s^{-1}{\mathrm{mW}}^{-1/2}\mathrm{cm}$ , the diffusion time constant τ=0.2 s , and the exposure energy change range is $0\sim 100\;\mathrm{mW}/{\mathrm{cm}}^{2}$ . It can be seen from the Figure 2 that as the exposure energy increases, the refractive index modulation gradually increases. With the recording light intensity increases, the saturation refractive index modulation gradually decreases. The greater the light intensity, the faster the refractive index modulation increases over time; that is, the exposure sensitivity increases.

**Figure 3:**
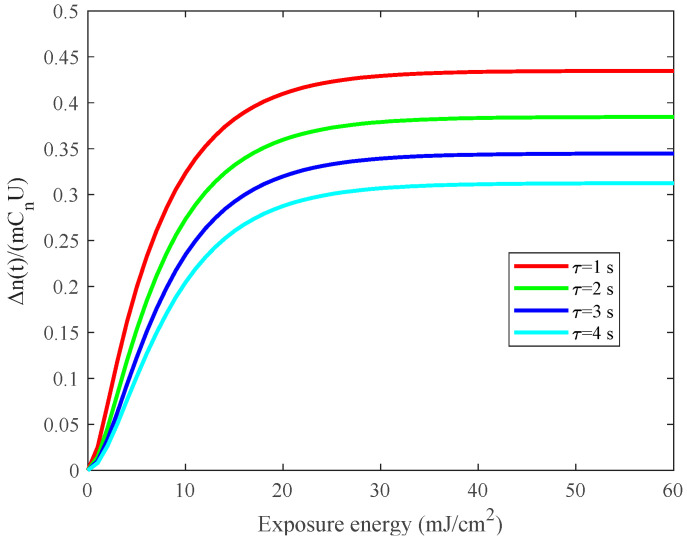
Analysis of factors affecting refractive index modulation: diffusion time constant.

It can be seen from the Figure 3 that as the diffusion time constant increases, the saturation refractive index modulation gradually decreases. Since the diffusion time constant $\tau =1/Dk^{2}$ , k=2π·SF , and *D* is a constant, the diffusion time constant is determined by the spatial frequency. The greater the spatial frequency, the smaller the diffusion time constant, which ultimately leads to an increase in the saturation refractive index modulation. As the spatial frequency increases, the fringe spacing decreases, and the distance of monomer diffusion becomes shorter, which makes the monomer polymerization reaction in the bright fringe area faster, and the refractive index modulation increases rapidly. Therefore, in order to increase the saturation refractive index modulation, a larger grating spatial frequency is required.

## 3. Tests of Volume Holography

In Section 2, we analyzed the relationship between diffraction efficiency and refractive index modulation of reflective volume holograms. In order to improve the diffraction efficiency, we conducted a qualitative analysis of its influencing factors, including exposure time, exposure intensity, and diffusion time constant. We optimized the exposure parameters through the above theoretical analysis. In order to analyze the transmission spectrum and diffraction characteristics of the photopolymer, the following section will conduct the test experiment of the reflective volume holograms on a photopolymer substrate, focusing on the analysis of diffraction efficiency and the recording effect of 3D image.

We use CRT20 holographic film made by Litiholo, which is a photopolymer composite material. The holographic film consists of two layers, the upper layer is a cellulose triacetate film (TAC) protective layer with a thickness of about 60 μm; the lower layer is a photopolymer with a thickness of about 16 μm. The material has a relatively wide sensitive band, and is sensitive to the visible spectral wavelength within 440–680 nm. The material can be used to record reflective volume transmission and reflection volume holograms, with simple operation, and no need for subsequent heat treatment or wet treatment. The spectral diffraction efficiency can be greater than 95%, the spectral bandwidth is greater than 15 nm, and the spectral shift is about 8 nm [22]. The material can achieve greater diffraction efficiency through reflection holographic recording. The diffraction efficiency is calculated by measuring the transmittance with a spectrophotometer. The calculation equation is as follows [23]:

(12) $$\eta =\frac{T_{\max}-T_{\min}}{T_{\max}}\times 100\%,$$

where in $T_{\min}$ represents the minimum value of transmittance in the test band, and $T_{\max}$ represents the sum of the transmitted light intensity and the diffracted light intensity of the material. $\left(T_{\max}-T_{\min}\right)$ represents the diffracted light intensity of the material. $T_{\min}$ is the trough of the transmission spectrum curve, and the wavelength shift occurs due to the shrinkage of the holographic recording material and the change in refractive index [23]. 

**Figure 4:**
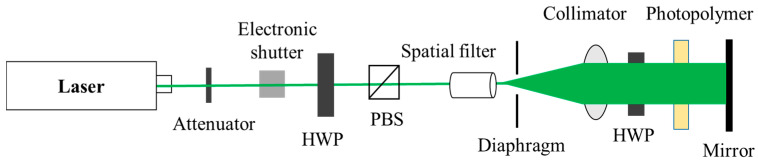
Vertical reflection type holographic recording optical setup.

Next, in order to test the diffraction efficiency of the material, as shown in Figure 4, a vertical reflective optical setup was used for interference processing. A laser is used for interference (532 nm), the laser is irradiated vertically on the surface of the photopolymer as the reference. Then, the laser beam that passes through the material is irradiated vertically on the mirror, and the light reflected by the mirror is irradiated vertically on the surface of the material as the object. The angle between the object light and the reference light is $180^{{}^{\circ}}$ , and the two beams interfere in the material. The used exposure light intensity is 1 mW/cm^2^, the exposure time is 4 min, the S polarized light is used for interference, the dark reaction is 4 min after the exposure, and the mercury lamp is irradiated for 2 min for curing.

**Figure 5:**
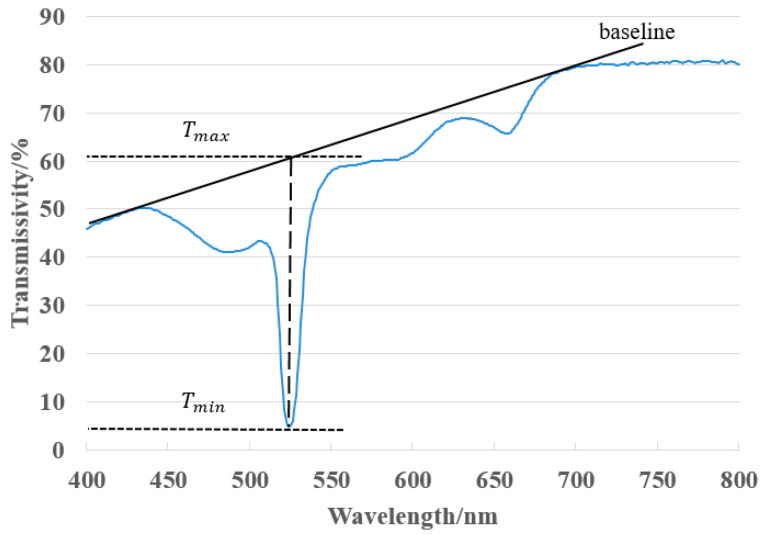
The transmission spectrum of the Litiholo holographic material after interference.

In order to measure the absorption characteristics of the photopolymer, the transmission spectrum of the material was measured with a spectrophotometer. The model of the spectrophotometer is U-4100 Spectrophotometer (Liquid), the scanning range is from 400 to 800 nm, and the scanning speed is 300 nm/min. The cured material was placed in a spectrophotometer to measure the transmission spectrum of the material, and the experimental results are shown in Figure 5. As shown in Figure 5, the baseline in the figure is used to approximate the transmittance of the bleached sample without holographic recording. $T_{\max}$ is expressed by the ordinate of the intersection of abscissa of trough and the baseline.

It can be seen from Figure 5 that the transmittance of the volume holographic grating near the 532 nm position is the smallest, at the trough position of the entire curve, and the transmittance at this time is 4.8%. The light transmittance of laser light deviating from 532 nm in the material will increase sharply, and the diffraction efficiency will drop sharply, which shows the wavelength selectivity of volume holographic gratings. In the entire curve, the maximum light transmittance is about 61%. According to Equation (12), the diffraction efficiency is 92%. This shows that volume holographic gratings have high diffraction efficiency.

**Figure 6:**
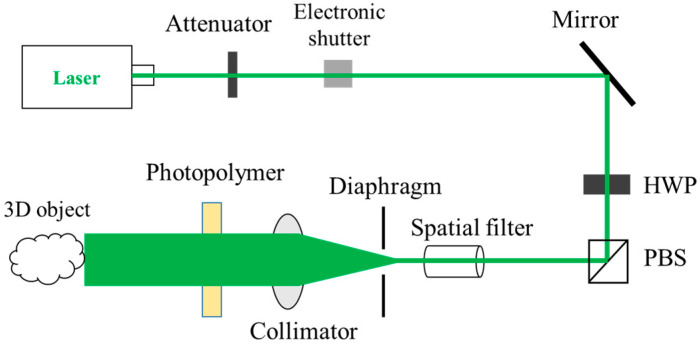
Monochromatic reflective volume holographic recording optical setup.

As shown in Figure 6, in order to test the 3D reconstruction effect of the volume holography, we built a monochromatic reflective volume holographic recording optical setup. A 3D physical object (piggy, 27 mm × 27 mm × 34 mm) is used for interference recording, and a green longitudinal mode laser (532 nm) is used as the coherent light source. The experimental procedure consists of three stages, which are coherent light interference recording, dark reaction, and mercury lamp irradiation curing.

**Figure 7:**
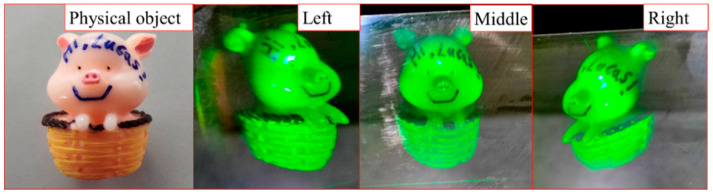
Volume holography reproduces monochromatic 3D objects under white light.

The reference light is irradiated on the surface of the object through the photopolymer, and the object light is generated by reflected diffusion. The angle between the reference light and the object light is 180°. The exposure intensity of the reference light is 1 mW/cm^2^, the exposure time is 4 min, the dark reaction time is 4 min, and the mercury lamp curing time is 2 min. The recontruction results of the recorded hologram under white light illumination are shown in Figure 7.

The recorded volume hologram can clearly reproduce the 3D piggy under the illumination of white light. Observed from three different angles on the left, middle, and right, the reconstructed images all have obvious depth information. It can be observed that the text information, “Hi, Lucas!”, in the reconstructed image, which has high resolution and good 3D display effect. As the volume hologram has wavelength selectivity, the illuminated light with a wavelength band that deviates from the recording wavelength will experience a sharp drop in diffraction efficiency. Therefore, the virtual image observed in the photopolymer is green. In addition, due to the angular selectivity of the volume hologram, it needs to be illuminated at a specific angle (the direction of the original reference light). The angle deviation will cause a sharp decrease of the diffraction efficiency. Measured by a spectrophotometer, the diffraction efficiency is 80%, indicating that the monochromatic reflective volume hologram display has a high diffraction efficiency.

## 4. Experimental Results and Discussion

**Figure 8:**
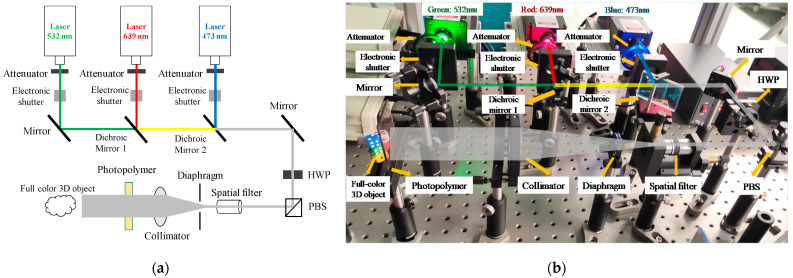
Construction of the RGB multiplexing optical setup: (**a**) schematic diagram of the interference optical setup; (**b**) physical diagram of the interference optical setup.

The above experiments prove that the photopolymer has good volume holographic diffraction performance. According to the principle of three primary colors, most monochromatic lights can be synthesized in different proportions with the three primary colors of RGB. In order to achieve full-color 3D recording, we designed the RGB multiplexing optical setup. Figure 8 shows the schematic diagram and physical diagram of the optical setup.

The lasers used in this research are the single longitudinal mode lasers of Changchun New Industry Optoelectronics Technology Co., Ltd., which are blue light (473 nm), green light (532 nm), and red light (639 nm). The coherence length of the three lasers is greater than 10 m, the spectral line width is less than 0.00004 nm, the beam divergence angle (full angle) is less than 3 mrad, with single longitudinal mode and continuous wave (CW). After the alignment inspection of the RGB multiplexing optical setup, it is found through experiments that when the exposure intensity is 300 $\mu W/{\mathrm{cm}}^{2}$ for red light, 1800 $\mu W/{\mathrm{cm}}^{2}$ for green light, and 900 $\mu W/{\mathrm{cm}}^{2}$ for blue light, we can synthesize better quality white light.

**Figure 9:**
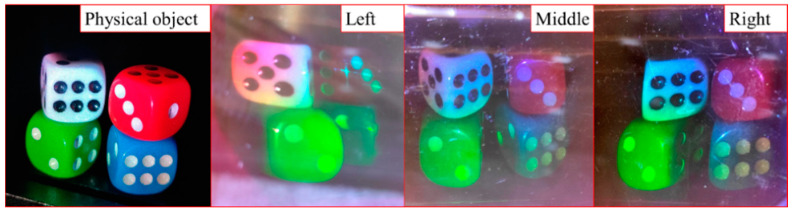
Volume holography-based RGB multiplexing full-color 3D display experimental results.

We conduct full-color 3D recording experiments through RGB simultaneous exposure. The dice (single size is 12 mm × 12 mm × 12 mm) are used as the actual object for interference recording. Three laser beams are controlled to be turned on and off at the same time through an electronic shutter. The exposure time is 2 min, and the total exposure energy is 360 mJ/cm^2^. The polymer is placed in darkness for 4 min, and finally cured with a mercury lamp for 2 min. It can be seen from Figure 9 that simultaneous exposure can achieve full-color 3D display. The photopolymer can clearly reproduce four dice of different colors under the irradiation of white light. We observe from three different angles on the left, middle, and right, and different sides of the dice can be observed from different angles. The observing angle of volume holography is about 90°. The colors of the four dice in the picture are consistent with the actual ones.

**Figure 10:**
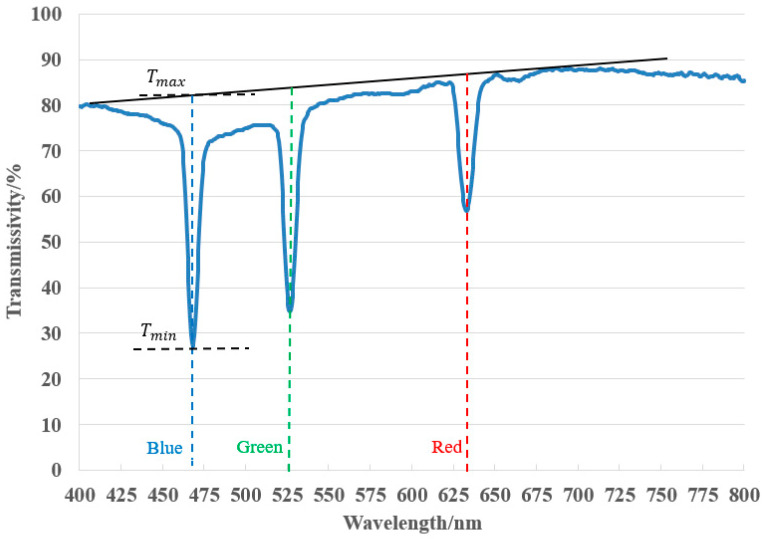
Transmission spectrum of full-color volume holography.

Since the object light is generated by the diffuse reflection of the surface, the light intensity of the object light will change with the various of position, resulting in different diffraction efficiency of the photopolymer at different positions. In order to calculate the diffraction efficiency more accurately, we replaced the dice with a mirror to make the object light more uniform, and ultimately ensure that the diffraction efficiency of the photopolymer surface is equal everywhere. Then, we use the RGB multiplexed optical setup in Figure 8 for interference recording. During the experiment, the real object was replaced with a mirror. The optical setup, experiment parameters and operation remained the same, and full-color volume holography was obtained through simultaneous exposure. After the volume holographic recording is completed, the transmission spectrum of the photopolymer hologram is measured with a spectrophotometer, as shown in Figure 10.

It can be seen from Figure 10 that the simultaneous exposure can achieve higher diffraction efficiency. After calculation by Equation (12), diffraction efficiency of 66.81% for recording wavelength in 473 nm, 58.34% for 532 nm and 34.08% for 639 nm were achieved. Blue light has the highest efficiency, followed by green light, and red light has the lowest diffraction efficiency. The diffraction efficiency for both blue and green light exceeds 50%, and good results have been achieved. The diffraction efficiency of red light is relatively low. This is due to the low intensity of red light in this experiment. The exposure intensity of red light is the smallest, and the exposure energy of red light is the smallest, so red light has the lowest diffraction efficiency. It can be seen from the transmission spectrum that there is a slight wavelength shift (approximately 8 nm) at the position of $T_{\min}$ , which is caused by the shrinkage and refractive index change of the photopolymer in the holographic recording. The volume holographic grating generated by simultaneous exposure recording has a high diffraction efficiency, with an average diffraction efficiency of 53.08%. In addition to the simultaneous exposure scheme, there is another way of sequential exposure. Although sequential exposure can reduce crosstalk [14], the simultaneous exposure scheme can shorten the overall exposure time and speed up the production efficiency of holograms. In addition, in the actual process of making holograms, for the scheme of sequential exposure, the overall exposure time becomes longer, and the optical setup is easily affected by the vibration of the platform, which will reduce the diffraction efficiency. 

Due to the limitation of equipment, the power of the blue laser used in this experiment is relatively small. After shaping by an attenuator, a dichroic mirror and a diaphragm, the maximum beam intensity irradiated on the surface of the photopolymer is only about 900 μW/cm^2^. This limits the maximum beam intensity of the RGB three-color laser. In this experiment, the exposure intensity is 300 μW/cm^2^ for red light, 1800 μW/cm^2^ for green light, 900 μW/cm^2^ for blue light, and the total exposure energy is 360 mJ/cm^2^. If high-power lasers are employed and the intensity of each laser is increased by 10 times, the recording time will be shortened by 10 times, and the exposure time can be reduced to 12 s. Therefore, the light intensity can be increased according to the requirement to shorten the exposure time. Since this experiment is to obtain higher diffraction efficiency, dark reaction and curing operations are augmented. In most practical applications, these two steps can also be omitted. In summary, we can greatly reduce the exposure time by increasing the light intensity.

According to Bragg’s law, the spatial frequency SF of the grating formed by blue, green and red light at a specific angle can be calculated. For reflective volume holography, when the Bragg angle is 90°, the volume holographic grating can obtain the maximum spatial frequency, $SF_{473nm}=6342\;\mathrm{lp}/\mathrm{mm},SF_{532nm}=5639\;\mathrm{lp}/\mathrm{mm},SF_{639nm}=4695\;\mathrm{lp}/\mathrm{mm}$ . The largest spatial frequency means that the diffraction efficiency and refractive index modulation of volume holography can be improved. According to Kogelnik’s coupled wave theory, we can calculate the relevant parameters of the 3D volume holography in the simultaneous exposure scheme. It can be seen from Table 1 that the average diffraction efficiency is 53.08%, and the average refractive index modulation is 0.01. The smallest spatial frequency is 4695 lp/mm, indicating that the grating fringe pitch is less than 0.3 μm, which improves the resolution and diffraction efficiency of the volume holography. 

The experimental results show that the full-color 3D image can be recorded by using the RGB multiplexed laser optical setup, and with only a single layer of photopolymer film. The reproduced image has high resolution (grating pitch less than 0.3 μm), strong three-dimensionality (depth of field greater than 10 mm), and large observing angle (about 90°). The volume holography has a compact structure and light weight, which realizes full-color 3D display through one single layer of photopolymer. 

**Figure 11:**
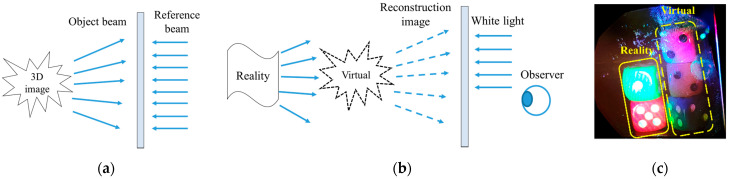
Recording and reconstruction procedure of volume holography: (**a**) volume holographic recording; (**b**) volume holographic reconstruction; (**c**) virtual and reality superposition.

Figure 11 shows the recording and reconstruction procedure of volume holography. As shown in Figure 11a, we can generate object light by diffuse reflection. Subsequently, the object light interferes with the reference light to record the wavefront information in the photopolymer. Using physical interference recording volume holograms avoids the limitations of SLM’s spatial bandwidth product, the number of pixels and diffraction angles, therefore it can reconstruct high-resolution 3D image. As shown in Figure 11b, under the illumination of white light, the observer can observe the scene where the real object and the virtual object are superimposed through the photopolymer. As the volume hologram has angle and wavelength selectivity, the reconstruction light in the real environment does not meet the Bragg condition and passes through the photopolymer directly. In Figure 11c, the image in the solid frame is the real scene, and the image in the dashed frame is the virtual scene, which shows that the combination of virtual and real is realized by a volume hologram. The proposed structure realized a full-color, high resolution, and large perspective 3D volume holographic display, which has good application prospects in augmented reality.

## 5. Conclusions

In this research, we propose a full-color see-through 3D display method based on volume holography. This method is based on real object interference, avoiding the device limitation of spatial light modulator (SLM). The volume holography has a light structure and weight, which realizes 3D display through one single film of photopolymer. For reflective volume holography, when the Bragg angle is 90°, the volume holographic grating can obtain the maximum spatial frequency, which means that the diffraction efficiency and refractive index modulation of volume holography can be improved. Experimental results show that the simultaneous exposure can achieve color holographic recording. The observing angle of the reconstructed image is about 90°, the average diffraction efficiency is 53.08%, and the grating fringe pitch is less than 0.3 μm. The reconstructed image of volume holography has high resolution, strong stereo perception, and large observing angle. Holographic 3D display can reproduce true 3D scenes and is currently one of the most promising 3D display technologies. The volume holographic full-color 3D display method proposed in this paper provides a basic technical reference and potential method for the practical application of augmented reality. 

## Figures and Tables

**Table 1:** 3D volume holography related parameters in the simultaneous exposure scheme.

Wavelength	Exposure Energy (mJ/cm^2^)	SF (lp/mm)	η (%)	Δn
B (473 nm)	108	6342	66.81	0.0108
G (532 nm)	216	5639	58.34	0.0106
R (639 nm)	36	4695	34.08	0.0085

## Data Availability

The data presented in this study are available on request from the corresponding author.
