# Correction: Wu et al. Full-Color See-Through Three-Dimensional Display Method Based on Volume Holography. *Sensors* 2021, *21*, 2698

**DOI:** 10.3390/s22030801

**Published:** 2022-01-21

**Authors:** Taihui Wu, Jianshe Ma, Chengchen Wang, Haibei Wang, Ping Su

**Affiliations:** 1 Department of Precision Instrument, Tsinghua University, Beijing 100084, China; wuth18@mails.tsinghua.edu.cn (T.W.); chengche19@mails.tsinghua.edu.cn (C.W.); 2 Tsinghua Shenzhen International Graduate School, Tsinghua University, Shenzhen 518055, China; ma.jianshe@sz.tsinghua.edu.cn (J.M.); whb20@mails.tsinghua.edu.cn (H.W.)

The authors make the following corrections to the published paper [1].

**Changes in Section 1. Introduction**

In paragraph 2, the sentence “Takeshi Yamaguchi et al. [10] designed a volume holographic printer to record 3D object images, segment 3D objects through multiple SLMs, and use 4f optical system to improve image reconstruction quality.” should be changed to “Takeshi Yamaguchi et al. [10] designed a volume holographic printer to record 3D object images, segment 3D objects through multiple computer-generated holograms, and use 4f optical system to improve image reconstruction quality.”

In paragraph 3, the sentence “The thickness of the holographic display film is only 16 μm, which can be applied to the next generation of integrated displays.” should be changed to “The thickness of the photopolymer is about 16 μm, which can be applied to the next generation of integrated displays.”

**Changes in Section 2.1. Coupled Wave Theory**

In paragraph 2, the sentence

ϕ is the tilt angle of the grating. Sorting out the above equations, the refractive index modulation of the volume holographic grating when the Bragg condition is satisfied can be calculated by the diffraction efficiency:

(6) $$\Delta n=\frac{\lambda \sqrt{\mathrm{arctanh}\left(\eta_{R}\right)}\sqrt{\cos\theta_{r}\cos\theta_{s}}}{\pi d},$$

should be changed to

ϕ is the tilt angle of the grating vector. Sorting out the above equations, the refractive index modulation of the volume holographic grating when the Bragg condition is satisfied can be calculated by the diffraction efficiency:

(6) $$\Delta n=\frac{\lambda \mathrm{arctanh}\left(\sqrt{\eta_{R}}\right)\sqrt{\cos\theta_{r}\cos\theta_{s}}}{\pi d},$$

In paragraph 3, the sentence “When the refractive index modulation is greater than 0.01, the diffraction efficiency of each wavelength exceeds 70%. When the refractive index modulation is greater than 0.03, the diffraction efficiency of each wavelength is close to 100%.” should be changed to “When the refractive index modulation is greater than 0.01, the diffraction efficiency of each wavelength exceeds 40%. When the refractive index modulation is greater than 0.04, the diffraction efficiency of each wavelength is close to 100%.”

In the original article, due to the inaccurate setting of the simulation parameter, there was a mistake in Figure 1 as published. The corrected Figure 1 appears below.

**Changes in Section 2.2. Monomer Diffusion Model**

In paragraph 3, the sentence

δ is the order of light response. $I_{0}$ is the exposure intensity. $\tau =1/Dk^{2}$ represents the diffusion time constant. *D* is the diffusion coefficient and κ is the polymerization coefficient, which are constants related to the material. When t→∞, that is, when the recording time is long enough, the above equation is simplified, and the saturation refractive index modulation of the material can be obtained:

(11) $$n_{SAT}=\frac{mC_{n}U}{\delta \left(\kappa I_{0}^{\delta }\tau +1\right)}.$$

should be changed to

δ is the order of magnitude of light response. $I_{0}$ is the exposure intensity. $\tau =1/Dk^{2}$ represents the diffusion time constant. *D* is the diffusion coefficient and κ is the polymerization coefficient, which are constants related to the material. When t≫τ, that is, when the recording time is long enough, the above equation is simplified, and the saturation refractive index modulation of the material can be obtained:

(11) $$\Delta n_{SAT}=\frac{mC_{n}U\delta }{\left(\kappa I_{0}^{\delta }\tau +1\right)}.$$

**Changes in Section 3. Tests of Volume Holography**

In paragraph 3, the sentence “The used exposure light intensity is, the exposure time is 4 min, the S polarized light is used for interference, the dark reaction is 4 min after the exposure, and the mercury lamp is irradiated for 2 min for curing.” should be changed to “The used exposure light intensity is 1 mW/cm^2^, the exposure time is 4 min, the S polarized light is used for interference, the dark reaction is 4 min after the exposure, and the mercury lamp is irradiated for 2 min for curing.”

In paragraph 4, the sentence “$T_{max}$ is expressed by the ordinate of the intersection of $T_{min}$ and the baseline.” should be changed to “$T_{\max}$ is expressed by the ordinate of the intersection of abscissa of trough and the baseline.”

**Changes in Section 4. Experimental Results and Discussion**

In paragraph 3, the sentence “The exposure is 4 min, and the total exposure energy is 360 mJ/cm^2^.” should be changed to “The exposure time is 2 min, and the total exposure energy is 360 mJ/cm^2^.”

In paragraph 8, the sentence “Compared with the previous work [23–25], it can be seen from Table 2 that the structure proposed in this paper has a higher average diffraction efficiency” needs to be deleted.

In the original article, Table 2 is not rigorous enough, and is not under the same standard or the same experimental parameters. Therefore, Table 2 needs to be deleted.

**Changes in References**

Due to the changes of Section 4, references [24,25] need to be deleted correspondingly.

The authors apologize for any inconvenience caused and state that the scientific conclusions are unaffected. The original article has been updated.

## Figures and Tables

**Figure 1:**
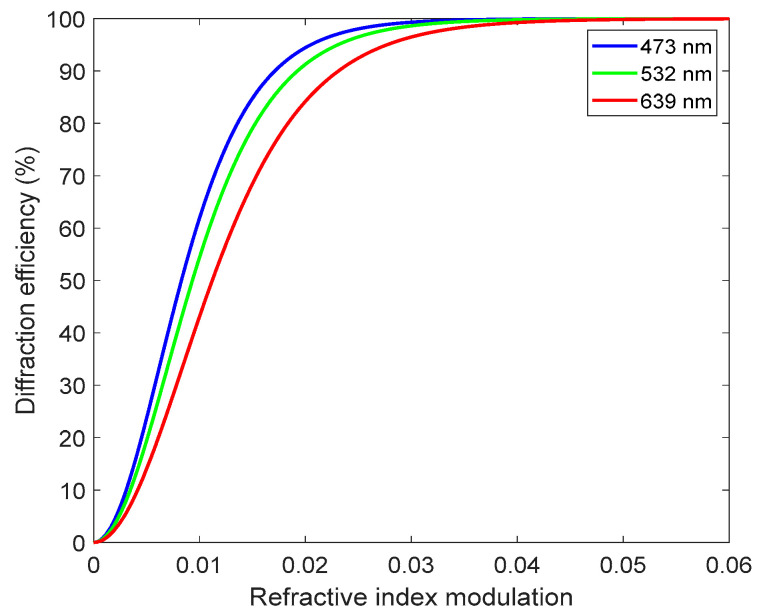
Reflective volume holographic grating: the relationship between refractive index modulation and diffraction efficiency.
